# Editorial: Technological innovations in neuropsychiatric disorders: virtual reality and beyond

**DOI:** 10.3389/fnhum.2026.1981276

**Published:** 2026-09-14

**Authors:** Júlio Belo Fernandes, Rafael A. Bernardes, Amanda C. Glueck, Sandeep Kumar Singh

**Affiliations:** 1 Egas Moniz Center for Interdisciplinary Research (CiiEM), Egas Moniz School of Health and Science, Almada, Portugal; 2 Nurs* Lab, Almada, Portugal; 3 Nursing Research Innovation and Development Centre of Lisbon (CIDNUR), School of Nursing of the University of Lisbon (ESEUL), Lisbon, Portugal; 4 Department of Neurology, College of Medicine, University of Kentucky, Lexington, KY, United States; 5 Department of Medical Biotechnology, All India Institute of Medical Sciences (AIIMS) Nagpur, Nagpur, India

**Keywords:** adaptive intervention, augmented reality, dementia, digital phenotyping, insomnia, outcome measurement, person-centered practice

## 1 Introduction

Much of the literature on digital technology in neuropsychiatry asks a single question: Does this technology work? The four contributions gathered in this Research Topic suggest a second question of comparable importance: What has technology allowed us to observe that conventional clinical encounters do not, and does this enable us to act early enough to benefit the patient? Read together, they shift the discussion away from virtuality and immersion toward temporal responsiveness: the capacity to detect change, interpret it as part of a trajectory, and adapt care accordingly. On this reading, “beyond” does not name the technology that succeeds virtual reality. This is called beyond episodic observation.

Zhang et al. combine an augmented-reality learning environment with concurrent electroencephalogram (EEG), ECG and eye-tracking in children with neurodevelopmental disorders. The most instructive findings are not the 89.3% classification accuracy but two temporal ones, and both come from a nested validation rather than the main cohort. In 18 children, 67.3% of adjustments in the physiologically adaptive condition were anticipatory, compared with 8.4% in the therapist-led behavioral observation condition. In the video analysis of 30 randomly selected state-change episodes, physiological signals preceded observable behavioral manifestations by a mean of 41 seconds. Therefore, the clinically relevant innovation is less the augmented environment than the shortening of the interval between change, detection, and response. The 12-month phase was single-arm; therefore, the longitudinal gains cannot be causally attributed to the intervention. These short-timescale findings come from a counterbalanced within-subjects comparison and are a separate matter.

Che et al. make a comparable move on a different clock. In a retrospective cohort of 2,271 patients receiving digital cognitive behavioral therapy for insomnia, daytime sleepiness rose early in treatment and peaked at week 2, which is an early signal that can be read as an adverse effect and encourage prompt discontinuation. However, week-2 exacerbation independently predicted a greater reduction in Pittsburgh Sleep Quality Index scores at weeks 4, 8, 12, and 16, after adjusting for sex, age, disease duration, education, and baseline severity. A signal that appears unfavorable at one time point acquires a different meaning when read as part of a trajectory. The study is retrospective, single-center, and based on self-report; adherence was not quantified. Even so, the practical implication is immediate, since what a clinician can say to a patient who wants to stop in the second week depends on which of the two readings is available.

Papamichael et al. supply the counterpoint this field most needs. Pooling nine randomized trials and 717 participants with dementia, gamified exergaming improved global cognition on the MoCA (MD 3.83; 95% CI 1.04–6.61) but showed no significant effect on the MMSE, activities of daily living, or the Short Physical Performance Battery. These findings should not be read simply as evidence of no benefit; the pooled MMSE estimate remained imprecise (MD 1.31; 95% CI −0.08 to 2.70) and the motor outcome was highly heterogeneous (*I*^2^ = 93.5%). Eight of the nine trials used non-immersive systems; advanced dementia was not represented among the trials reporting disease stage; and active gameplay time was inconsistently documented relative to the total session duration. Whether, greater immersion adds therapeutic value in dementia remains insufficiently tested: the evidence in traumatic brain injury has been mixed (Aulisio et al., 2020), and newer formats such as mixed reality have also been explored in small clinical series (Medina et al., 2025). In short, technological resolution does not guarantee evaluative resolution. The field risks developing interventions that change continuously while evaluating them episodically.

Conca et al. offer language for why this is important. Their Perspective reframes psychiatric illness as a crisis of regulation and meaning arising within dynamically interacting multilevel networks, drawing from synergetics the notions of attractor states, instability, and *kairos*—the narrow window in which a small, well-timed perturbation can reorganize a system. If neuropsychiatric states are dynamic rather than static, clinically significant information lies not only in the states themselves but also in the transitions between them. Digital phenotyping offers one route to the empirical operationalization of such windows. The authors are equally careful about the corollary: artificial intelligence augments clinical judgement rather than directing it.

Two consequences follow, which run in different directions. Where observation becomes increasingly continuous, evaluation remains episodic, and the mismatch limits what an evaluation can register. Interventions that adapt also require designs capable of accommodating repeated adaptation; microrandomized offer an approach to estimating proximal effects across repeated decision points (Klasnja et al., 2015). However, the evaluation can be too narrow or coarse. A framework analysis of 21 randomized trials of technology-based gait rehabilitation found that tailoring training parameters was largely a by-product of clinical pragmatism or device functionality rather than a deliberate, person-centered process, and no single trial included a patient-reported experience measure (Fernandes et al., 2026). Higher resolution is not self-justifying: personalization built on increasingly granular data is not the same as person-centered care (Fernandes and McCormack, 2026). A third question sits beneath both: are the mechanisms invoked to explain these effects being measured directly or inferred from the outcomes they are meant to explain?

The burden of higher-resolution measurements is not evenly distributed. In Zhang et al., 51.1% of children with autism initially avoided the EEG cap, compared with 7.9% of those with ADHD; graded desensitization enabled 21 of the 23 initially avoidant children to proceed with the EEG test. The authors further estimated, for implementation planning, that roughly 1 in 10 interested families may remain unable to tolerate such procedures, despite accommodation. The burden of this kind is an argument for treating usability, accessibility, and user experience as design questions rather than limitations reported after the fact (Bernardes et al., 2025).

The promise of these technologies may, therefore, depend less on how immersive they become than on whether observation, interpretation, and response can be brought close enough together to change what happens next—and on whether the person remains visible at that resolution.
